# Comparative Genomics and Phylogenomic Analysis of the Genus *Salinivibrio*

**DOI:** 10.3389/fmicb.2019.02104

**Published:** 2019-09-11

**Authors:** Rafael R. de la Haba, Clara López-Hermoso, Cristina Sánchez-Porro, Konstantinos T. Konstantinidis, Antonio Ventosa

**Affiliations:** ^1^Department of Microbiology and Parasitology, Faculty of Pharmacy, University of Seville, Seville, Spain; ^2^School of Civil and Environmental Engineering, Georgia Institute of Technology, Atlanta, GA, United States

**Keywords:** *Salinivibrio*, *Salinivibrio kushneri*, complete genome, phylogenomics, genomics, synteny, halophilic bacteria, hypersaline environments

## Abstract

In the genomic era phylogenetic relationship among prokaryotes can be inferred from the core orthologous genes (OGs) or proteins in order to elucidate their evolutionary history and current taxonomy should benefits of that. The genus *Salinivibrio* belongs to the family *Vibrionaceae* and currently includes only five halophilic species, in spite the fact that new strains are very frequently isolated from hypersaline environments. Species belonging to this genus have undergone several reclassifications and, moreover, there are many strains of *Salinivibrio* with available genomes which have not been affiliated to the existing species or have been wrongly designated. Therefore, a phylogenetic study using the available genomic information is necessary to clarify the relationships of existing strains within this genus and to review their taxonomic affiliation. For that purpose, we have also sequenced the first complete genome of a *Salinivibrio* species, *Salinivibrio kushneri* AL184^T^, which was employed as a reference to order the contigs of the draft genomes of the type strains of the current species of this genus, as well as to perform a comparative analysis with all the other available *Salinivibrio* sp. genomes. The genome of *S. kushneri* AL184^T^ was assembled in two circular chromosomes (with sizes of 2.84 Mb and 0.60 Mb, respectively), as typically occurs in members of the family *Vibrionaceae*, with nine complete ribosomal operons, which might explain the fast growing rate of salinivibrios cultured under laboratory conditions. Synteny analysis among the type strains of the genus revealed a high level of genomic conservation in both chromosomes, which allow us to hypothesize a slow speciation process or homogenization events taking place in this group of microorganisms to be tested experimentally in the future. Phylogenomic and orthologous average nucleotide identity (OrthoANI)/average amino acid identity (AAI) analyses also evidenced the elevated level of genetic relatedness within members of this genus and allowed to group all the *Salinivibrio* strains with available genomes in seven separated species. Genome-scale attribute study of the salinivibrios identified traits related to polar flagellum, facultatively anaerobic growth and osmotic response, in accordance to the phenotypic features described for species of this genus.

## Introduction

Firstly described by Mellado et al. (1996) to accommodate the species *Vibrio costicola* (Smith, 1938), members of the genus *Salinivibrio*, belonging to the family *Vibrionaceae*, class *Gammaproteobacteria*, have been isolated from diverse habitats, such as aquatic hypersaline systems, brines, salted meats, and saline soils (Chamroensaksri et al., 2009; Gorriti et al., 2014; Ventosa, 2015; López-Hermoso et al., 2018a, b). The taxonomic affiliation of the type and other 70 representative strains of this genus has been recently evaluated using a Multi Locus Sequence Analysis (MLSA) (López-Hermoso et al., 2017b, 2018a). Furthermore, draft genomic sequences of the type strains and only a few (14) representative strains has also been used to delineate *Salinivibrio* species (López-Hermoso et al., 2018a, b). As a result of the aforementioned studies, the genus *Salinivibrio* is currently composed of five species: *S. costicola* [containing two subspecies, i.e., *S. costicola* subsp. *costicola* (Garcia et al., 1987; Mellado et al., 1996) and *S. costicola* subsp. *alcaliphilus* (Romano et al., 2005)], *Salinivibrio kushneri* (López-Hermoso et al., 2018b), *S. proteolyticus* [which also includes the former *S. costicola* subsp. *vallismortis* (Amoozegar et al., 2008b; López-Hermoso et al., 2018a)], *S. sharmensis* (Romano et al., 2011), and *Salinivibrio siamensis* (Chamroensaksri et al., 2009). Additionally, the species “*Salinivibrio socompensis*” has also been proposed to include three *Salinivibrio* strains (Gorriti et al., 2014), but this name has not been validly published.

Although forty-six *Salinivibrio* genomes are available in GenBank database, all of them represent draft genomes and no complete genome projects have been conducted within this genus. Besides, only two studies dealing with genome sequence analysis of salinivibrios have been published (Gorriti et al., 2014; López-Hermoso et al., 2017a), but those were focused on a few genomes (the former) or the analysis only provided genome statistics (the latter), and in both cases using draft genome sequences. On the other hand, almost half of the available *Salinivibrio* genomes are not classified at the species level or misnamed (wrongly designated). The relationship of *Salinivibrio* species is not totally clear or stable according to previous studies because phylogenetic trees were based on MLSA of only up to eight housekeeping genes (Gorriti et al., 2014; López-Hermoso et al., 2017b) or the phylogenomic analysis was conducted only with a few of the *Salinivibrio* genomes (López-Hermoso et al., 2018a, b).

In this study, we clarify the phylogenetic relationships of existing genomes and species and review the taxonomic affiliation of the strains included within the genus *Salinivibrio* using a wide phylogenomic approach. This work also reports the first complete genome of a species of the genus *Salinivibrio*, *S. kushneri*, which was used as a reference to order the contigs of the draft genomes of the type strains within this genus, as well as to perform a comparative analysis with all the other available *Salinivibrio* genomes.

## Materials and Methods

### Culture Conditions and Genomic DNA Extraction

The strain *S. kushneri* AL184^T^ was obtained from our culture collection where it was stored at −80°C and it was cultured in liquid SW broth (whose composition in g l^–1^ is: NaCl, 58.5; MgCl_2_^⋅^6H_2_O, 9.75; MgSO_4_^⋅^7H_2_O, 15.25; CaCl_2_, 0.25; KCl, 1.5; NaHCO_3_, 0.05; NaBr, 0.175; and yeast extract, 5.0) at 37°C with the pH adjusted between 7.2 and 7.4, according to López-Hermoso et al. (2018b). High-quality genomic DNA was extracted using the QIAmp DNA Mini Kit (Qiagen) following the manufacturer’s instructions.

### Genome Sequencing, Assembly, and Annotation

A single molecule real-time sequencing approach was accomplished by using PacBio technologies. For that purpose, a PacBio library with 10 kbp insert size was constructed and a 350× sequencing depth was achieved. Additionally, whole genome shotgun reads obtained from an Illumina HiSeq (2 × 100-bp paired-end reads) device in an earlier study (López-Hermoso et al., 2017a) were used to carry out a hybrid assembly using SPAdes v.3.13.0 (Bankevich et al., 2012). Subsequently, the final assembly was achieved using only filtered by quality PacBio reads (read length after trimming ≥5000 nt, polymerase read quality ≥0.80, and polymerase read length ≥100) using an Overlap-Layout-Consensus algorithm as implemented in the HGAP v.2 pipeline (Chin et al., 2013). Dot plots to check for circularity at contig ends were drawn using Gepard v.1.40 software (Krumsiek et al., 2007). The resulting genome was circularized using toAmos and minimus2 (Sommer et al., 2007), followed by Circlator (Hunt et al., 2015) tools.

All the forty-six *Salinivibrio* draft genomes available in GenBank database were recovered, with the exception of that of *S. costicola* subsp. *costicola* ATCC 33508^T^ (assembly accession no. GCA_000390145.1), which presented a suspicious length (4.78 Mb) and is excluded from RefSeq database due to its low quality sequence, and that of *S. kushneri* AL184^T^ (assembly accession no. GCA_001995845.1), which was replaced by the complete genome of this strain achieved in this study (Table 1). Automatic annotation of those draft genomes together to the complete genome of strain *S. kushneri* AL184^T^ achieved in this study was performed using RAST (Overbeek et al., 2014) and KAAS-KEEG (Moriya et al., 2007). RNA genes were determined by means of RNAmmer software (Lagesen et al., 2007).

**TABLE 1:** *Salinivibrio* genomes available in GenBank database used in this study, including their basic statistical information.

**New proposed strain designation (original strain label)**	**Assembly no.**	**Completeness level**	**Size (Mb)**	**GC (mol%)**	**Scaffolds**	**CDS**
*Salinivibrio costicola* subsp. *costicola* LMG 11651^T^	GCA_000565345.1	Contig	3.38	49.3	202	2332
*Salinivibrio costicola* subsp. *alcaliphilus* DSM 16359^T^	GCA_001996185.1	Contig	3.38	49.3	248	2949
*Salinivibrio costicola* AR640 (*Salinivibrio* sp. AR640)	GCA_001995405.1	Scaffold	3.26	49.3	60	2873
*Salinivibrio costicola* AR647 (*Salinivibrio* sp. AR647)	GCA_001995415.1	Contig	3.28	49.3	22	2920
*Salinivibrio costicola* IB643 (*Salinivibrio* sp. IB643)	GCA_001996105.1	Contig	3.12	49.4	91	2739
*Salinivibrio costicola* MA351 (*Salinivibrio* sp. MA351)	GCA_001996065.1	Contig	3.31	49.4	59	2923
*Salinivibrio costicola* MA427 (*Salinivibrio* sp. MA427)	GCA_001996115.1	Scaffold	3.26	49.5	513	2739
*Salinivibrio costicola* MA440 (*Salinivibrio* sp. MA440)	GCA_001996265.1	Contig	3.42	49.3	102	2996
*Salinivibrio costicola* MA607 (*Salinivibrio* sp. MA607)	GCA_001996245.1	Contig	3.35	49.2	68	2946
*Salinivibrio kushneri* AL184^T^	CP040021, CP040022	Complete	3.44	50.7	1	3055
*Salinivibrio kushneri* BNH (*Salinivibrio* sp. BNH)	GCA_001722105.1	Contig	3.48	50.5	45	3073
*Salinivibrio kushneri* HTSP (*Salinivibrio* sp. HTSP)	GCA_003390965.1	Scaffold	3.39	50.6	121	3007
*Salinivibrio kushneri* IB282 (*Salinivibrio* sp. IB282)	GCA_001995685.1	Contig	3.23	50.7	157	2836
*Salinivibrio kushneri* IB560	GCA_001995915.1	Contig	3.49	50.5	65	3081
*Salinivibrio kushneri* IB563	GCA_001995785.1	Scaffold	3.48	50.5	173	3031
*Salinivibrio kushneri* IC202	GCA_001995805.1	Contig	3.61	50.5	111	3160
*Salinivibrio kushneri* IC317	GCA_001995875.1	Scaffold	3.28	50.9	236	2881
*Salinivibrio kushneri* MA421	GCA_001995635.1	Scaffold	3.54	50.4	70	3112
*Salinivibrio kushneri* ML277	GCA_001995865.1	Contig	3.45	50.4	73	3036
*Salinivibrio kushneri* ML318	GCA_001995885.1	Scaffold	3.36	50.7	96	2978
*Salinivibrio kushneri* ML323 (*Salinivibrio* sp. ML323)	GCA_001995745.1	Contig	3.23	50.7	63	2865
*Salinivibrio kushneri* ML328A	GCA_001995725.1	Contig	3.39	50.6	70	3001
*Salinivibrio kushneri* ML331	GCA_001995995.1	Contig	3.55	50.5	112	3143
*Salinivibrio kushneri* PRJEB21454 (*Salinivibrio costicola* PRJEB21454)	GCA_900188555.1	Scaffold	3.32	50.5	23	2952
*Salinivibrio proteolyticus* DSM 19052^T^	GCA_001996165.1	Contig	3.6	49.8	51	3234
*Salinivibrio proteolyticus* DSM 8285	GCA_001996225.1	Contig	3.5	49.9	95	3130
*Salinivibrio proteolyticus* DV (*Salinivibrio* sp. DV)	GCA_001722075.1	Contig	3.71	49.8	145	3268
*Salinivibrio proteolyticus* IB574	GCA_001996205.1	Scaffold	3.61	49.8	69	3187
*Salinivibrio proteolyticus* IB872	GCA_001995345.1	Scaffold	3.64	49.8	102	3201
*Salinivibrio proteolyticus* PR5	GCA_001996085.1	Contig	3.46	50	105	3035
*Salinivibrio proteolyticus* PR919	GCA_001995355.1	Contig	3.49	49.9	176	3076
*Salinivibrio proteolyticus* PR932	GCA_001996125.1	Scaffold	3.5	49.9	74	3085
*Salinivibrio proteolyticus* YCSC6 (*Salinivibrio* sp. YCSC6)	GCA_002369825.1	Contig	3.71	49.8	2	3262
*Salinivibrio sharmensis* DSM 18182^T^	GCA_001995985.1	Contig	3.33	50.3	40	2944
*Salinivibrio siamensis* JCM 14472^T^	GCA_001996005.1	Contig	3.44	50.4	61	3025
*Salinivibrio siamensis* IB868 (*Salinivibrio* sp. IB868)	GCA_001995655.1	Contig	3.44	50.5	30	3049
*Salinivibrio siamensis* IB870 (*Salinivibrio* sp. IB870)	GCA_001995945.1	Contig	3.44	50.5	42	3036
*Salinivibrio siamensis* KP-1 (*Salinivibrio* sp. KP-1)	GCA_000968645.1	Contig	3.5	50.5	49	3110
*Salinivibrio siamensis* ML198 (*Salinivibrio* sp. ML198)	GCA_001996015.1	Contig	3.43	50.5	87	3015
*Salinivibrio siamensis* ML290 (*Salinivibrio* sp. ML290)	GCA_001995695.1	Scaffold	3.52	50.3	36	3128
*Salinivibrio siamensis* PR6 (*Salinivibrio* sp. PR6)	GCA_001995955.1	Contig	3.44	50.4	49	3055
“*Salinivibrio socompensis*” S10B	GCA_000565325.1	Contig	3.35	49.5	252	2310
“*Salinivibrio socompensis*” S34	GCA_000513735.1	Contig	3.33	49.4	334	ND
“*Salinivibrio socompensis*” S35	GCA_000513715.1	Contig	3.41	49.5	270	2311
*Salinivibrio* sp. ES.052	GCA_900141775.1	Contig	3.57	49.3	6	3146

*ND, not determined.*

### Synteny Analysis

Mauve genome alignment program (Darling et al., 2004) was employed to address the issue of genome rearrangements and contig ordering of the draft genomes by pairwise comparison using the complete genome of *S. kushneri* AL184^T^ as a reference. Progressive Mauve software (Darling et al., 2010) was used for multiple genome alignment of the previously ordered draft genomes together to the complete genome of the type strains within the genus in order to find locally collinear blocks (LCBs). Synteny among different genomes was determined by measuring the alignment length of all the LCBs shared between the genomes being compared.

Circular plots showing LCBs and links between *Salinivibrio* genomes were drawn with the use of Circos v.0.69, a circular visualization software (Krzywinski et al., 2009). Furthermore, this program was utilized to represent the large and small circular chromosomes of *S. kushneri* AL184^T^.

### Phylogenomic Analysis

All predicted protein-coding genes and proteins annotated from each available genome were searched using an all-vs.-all BLAST comparison using the Enveomics collection tools (Rodriguez-R and Konstantinidis, 2016). This analysis allows us to detect shared reciprocal best matches (described as those with equal or above 70% nucleotide identity or 40% amino acid identity) in all pairwise genome comparisons (core OGs or proteins) of the 45 *Salinivibrio* strains under study. The single copy core genes and proteins, respectively, were individually aligned using MUSCLE (Edgar, 2004). The resulting nucleotide and amino acids alignments were automatically trimmed using trimAl v.1.4 on “gappyout” mode (Capella-Gutiérrez et al., 2009) and, subsequently, concatenated to create core-genome and core-proteome alignments, and the phylogenomic trees were reconstructed by maximum-likelihood method with FastTreeMP v.2.1.8 (Price et al., 2010) where the branch support was estimated by means of the Shimodaira-Hasegawa test (Shimodaira and Hasegawa, 1999; Goldman et al., 2000).

Calculation of the orthologous average nucleotide identity (OrthoANI) among the studied genomes was accomplished employing USEARCH v8.1.1861 as implemented in OrthoANIu tool (Yoon et al., 2017). When genome pair comparisons showed less than 80% ANI values such genomes have divergent too much to use nucleotide level search and some genes might be missing; thus, average amino acid identity (AAI) was also determined. Mean AAI values for each genome pair were calculated using reciprocal best hits (two-way AAI) with the appropriate script in the Enveomics collection tools (Rodriguez-R and Konstantinidis, 2016).

### Metagenomic Analysis and Fragment Recruitment Plots

To detect putative novel *Salinivibrio* species using culture-independent approaches, four 16S rRNA gene amplicon datasets reporting the presence of salinivibrios in their respective environments (Tkavc et al., 2011; Zhang et al., 2016, 2017; Crisler et al., 2019) were obtained from GenBank and NCBI’s Sequence Read Archive databases (accession no. FN823320-FN824096, SRP072906, SRP090542, SRP089997, and SRP090529) or provided by the authors. For those amplicon data, read quality filter was performed using Prinseq (Schmieder and Edwards, 2011), and chimera detection and filtering, OTU’s picking (at 97% similarity clustering value), representative picking of each OTUs, and taxonomy assignment were achieved using the software QIIME v. 1.9.0 (Caporaso et al., 2010).

Abundance estimation of *Salinivbrio* type strains and close relatives in several hypersaline environments was carried out by means of fragment recruitment using shotgun metagenomic databases (Supplementary Table 1). To avoid analysis bias, contigs of each of the genomes were concatenated and, subsequently, the rRNA gene sequences were masked. Blastn search (with the following parameters: length of the alignment ≥ 30 nt, similarity >95%, E value ≤ 1e-5) was employed to map the metagenomic reads (previously filtered to assess their quality) against each genome. The top-best-hit recovered after Blastn search was used to construct the recruitment plots.

## Results and Discussion

### Complete Genome Sequencing of *Salinivibrio kushneri* AL184^T^

The species *S. kushneri* was proposed based on 10 isolates, with strain AL184^T^ designated as the type strain (López-Hermoso et al., 2018b). In despite of the fact that strains belonging to the genus *Salinivibrio* are commonly isolated due to their fast and easy growth on regular laboratory media, only a few species within this genus have been described so far. The relative low number of species is in spite the fact that different media and culture conditions (temperature, pH, salinity, and aerobic/anaerobic growth) were used over the time to isolate new *Salinivibrio* strains and to attempt to describe new species (Huang et al., 2000; Sánchez-Porro et al., 2003; Caton et al., 2004; Romano et al., 2005, 2011; Yeon et al., 2005; Amoozegar et al., 2008a, b; Zhu et al., 2008; Chamroensaksri et al., 2009; Xiao et al., 2009; Al-Mailem et al., 2014; Gorriti et al., 2014; Ashengroph, 2017; López-Hermoso et al., 2017b, 2018b; Le and Yang, 2018). In any case, there might be still cultural biases. Therefore, although the draft genome of the strain AL184^T^ was already published elsewhere (López-Hermoso et al., 2017a), given the potential interest that this new species might have to unveil the speciation processes within this genus and its ecological role, the complete genome sequence of strain AL184^T^ was obtained in this study.

**FIGURE 1:**
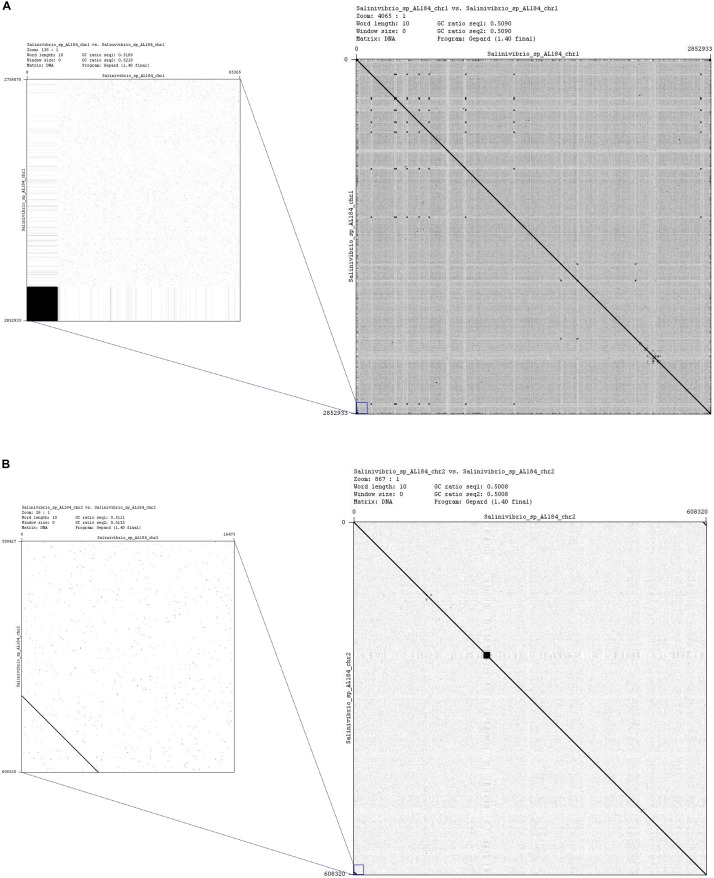
Dot plots displaying the comparison of assembled contig 1 **(A)** and contig 2 **(B)** of *Salinivibrio kushneri* AL184^T^ against itself. The enlarged images of the dot plots show that both ends of each contig are identical and, therefore, demonstrate that they constitute two circular chromosomes.

Firstly, a hybrid assembly by using PacBio and Illumina reads was performed with the aim of accurately estimating the genome size, which we determined to be 3,436,949 bp. This result was required to achieve the final assembly based on the PacBio reads. According to PacBio recommendations and to Chin et al. (2013), the PacBio-only *de novo* assembly is preferred when it is possible to get at least 50X coverage. For this strain, the sequencing depth obtained was 350×, which motivated the choice of a PacBio-only based assembly strategy. Not a single contig, but two were obtained after the assembly, which was expected since other members of the family *Vibrionaceae* have been reported to contain two chromosomes (Dikow and Smith, 2013; Bernardy et al., 2016; Rashid et al., 2016; Kachwamba et al., 2017). In order to test this hypothesis a dot plot of each contig was carried out to check overlapping between the ends (Figure 1). Both dot plots showed this overlapping between the start and the end of each contig, indicating that strain AL184^T^ contains two circular chromosomes. In addition, dot plot results were confirmed by BLAST search. Therefore, the two chromosomes were circularized and, subsequently, a linearized version was output with the *dnaA* gene as the starting position for chromosome I and a random gene for chromosome II. The empirical per-base coverage achieved was 211× for chromosome I and 216× for chromosome II.

**FIGURE 2:**
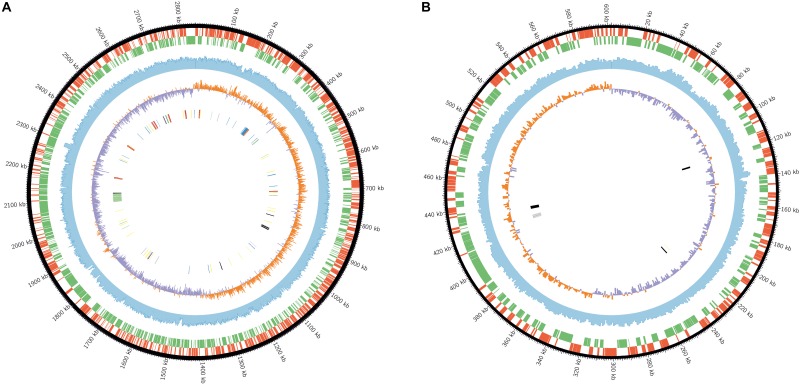
Graphical circular map of the chromosome I **(A)** and choromosome II **(B)** of *S. kushneri* AL184^T^. From the outer to inner chromosomal rings: (1) predicted CDSs transcribed in a clockwise direction; (2) predicted CDSs transcribed in a counterclockwise direction; (3) GC content in a 1,000-bp sliding window; (4) GC skew (C-G/G+C) in a 1,000-bp sliding window; (5) rRNAs (red), tRNAs (yellow), ribosomal proteins (blue), flagellum and flagellar motility genes (green), compatible solute synthesis genes (gray), compatible solute transporters (black), and anaerobic respiration-related genes (purple).

The final assembly for strain AL184^T^ consists of two circular chromosomes with 2,840,906 bp and 602,384 bp, respectively (Figure 2). The completeness and contamination of the genome (both chromosomes together) estimated by CheckM tool (Parks et al., 2014) was 99.9% and 0.54%, respectively, which means that virtually the complete genome with a negligible -if any- amount of contamination was recovered. Although no essential genes were found in chromosome II, several lines of evidence suggest that it is a chromosome rather than a plasmid: (i) DNA G+C content was very similar for both chromosomes (50.8 mol% for I and 50.1 mol% for II); (ii) the length of chromosome II is more likely to correspond to a chromosome instead of a plasmid; (iii) the existence of genes theoretically belonging to the same cluster in different chromosomes, for example the cluster *betABC*, involved in the synthesis of glycine betaine, was coded in chromosome I (genes *betA* and *betB*) and chromosome II (gene *betC*); and (iv) as aforementioned, the members of the family *Vibrionaceae* usually have two chromosomes (Dikow and Smith, 2013; Bernardy et al., 2016; Rashid et al., 2016; Kachwamba et al., 2017).

Annotation of the complete genome predicted 1244 and 233 CDS transcribed in a clockwise direction and 1280 and 298 CDS transcribed in a counter clockwise direction for chromosomes I and II, respectively (Figure 2). A total of 28 rRNA, 95 tRNA, 57 ribosomal protein, 51 flagellum and flagellar motility, 5 compatible solute synthesis, 10 compatible solute transporter, and 4 anaerobic respiration-related genes were identified in chromosome I, whereas only 1 tRNA, 4 compatible solute synthesis, and 5 compatible solute transporter genes were detected in chromosome II (Figure 2). This large number of rRNA genes, which were clustered in nine complete ribosomal operons (and an additional 5S rRNA gene located only 300 bp downstream with respect to one of the rRNA operons), might explain the fast growing rate of salinivibrios cultured in copiotrophic laboratory conditions (Roller et al., 2016). This finding was also observed in related taxa, such as *Vibrio cholerae*, that contains eight complete ribosomal operons (Rodicio and Mendoza, 2004) and it is a common trait of fast reproduction organisms (Roller et al., 2016). It is well-known that the different 16S rRNA genes contained in the same strain can be heterogeneous up to some extent; in the case of strain AL194^T^ the nine 16S rRNA genes presented 100–98.4% sequence similarity, which is, practically, within the cutoff value currently used for species delineation (Kim et al., 2014).

### Synteny Analysis Among *Salinivibrio* Type Strain Genome Assemblies

Analyses of conservation of homologous genes and gene order between two or more genomes of different species (synteny) play a pivotal role in comparative genomics (Lee et al., 2018) and can provide insights into evolutionary processes that lead to diversity, chromosomal dynamics, and rearrangement rates between species (Bhutkar et al., 2006). Although analysis of synteny among closely related species is now widely used for every new published genome, this analysis is regularly performed on assembled sequences that are fragmented, neglecting the fact that most methods were developed using complete genomes (Liu et al., 2018). Here, we have used the complete genome of *S. kushneri* AL184^T^ as a reference to reconstruct the fragmented genomes of the type strains of species of the genus *Salinivibrio* by ordering the contigs and assigning them to either chromosome I or II (Rissman et al., 2009). Following this strategy, chromosome I of the type species of the genus *Salinivibrio*, *S. costicola* subsp. *costicola* LMG 11651^T^, would be formed by 90 contigs (2,513,750 bp), and chromosome II by 39 contigs (691,570 bp), while the remaining 73 contigs (174,314) of that assembly could not be assigned to either chromosome I or II (Supplementary Figure 1 and Supplementary Table 2), and presumably represent gene-content differences between the two genomes compared. For the type strains of the other species or subspecies of the genus, chromosome I was constituted by 63 (2,545,420 bp), 32 (2,869,249 bp), 26 (2,717,996 bp), and 40 (2,729,566 bp) contigs corresponding to *S. costicola* subsp. *alcaliphilus* DSM 16359^T^, *S. proteolyticus* DSM 19052^T^, *S. sharmensis* DSM 18182^*T*^, and *S. siamensis* JCM 14472^T^, respectively, and the chromosome II of those strains was composed of 19 (706,389 bp), 10 (716,104 bp), 6 (589,195 bp), and 12 (688,170 bp) contigs, respectively, while 166 (129,848 bp), 9 (18,143 bp), 8 (19,704 bp), and 9 (24,449 bp) contigs left unassigned, respectively (Supplementary Figure 1 and Supplementary Table 2).

**FIGURE 3:**
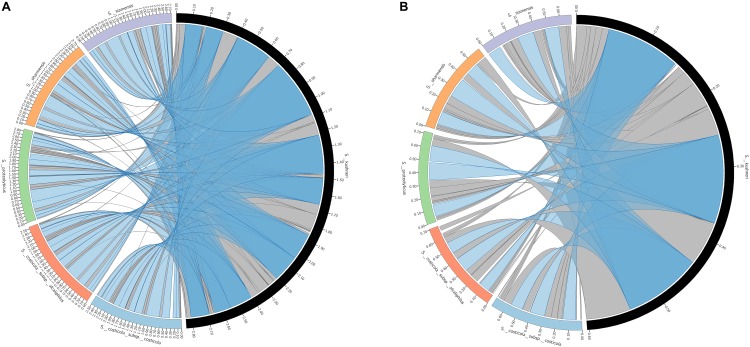
Pairwise alignment of locally collinear blocks (LCBs) between large **(A)** and small **(B)** chromosomes of *S. kushneri* AL184^T^ and those of *S. costicola* subsp. *costicola* LMG 11651^T^, *S. costicola* subsp. *alcaliphilus* DSM 16359^T^, *S. proteolyticus* DSM 19052^T^, *S. sharmensis* DSM 18182^T^, and *S. siamensis* JCM 14472^T^. Blue bands represents LBC > 100 Kb and gray bands LCB < 100 Kb.

**FIGURE 4:**
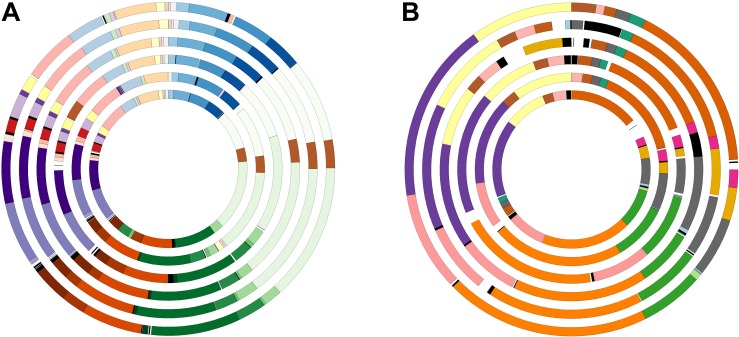
*Salinivibrio* large (87 LCBs) **(A)** and small (30 LCBs) **(B)** chromosomes circular plots. Each circle represents a genome. From the outer most circle: *S. kushneri* AL184^*T*^, *S. costicola* subsp. *costicola* LMG 11651^T^, *S. costicola* subsp. *alcaliphilus* DSM 16359^T^, *S. proteolyticus* DSM 19052^T^, *S. sharmensis* DSM 18182^T^, and *S. siamensis* JCM 14472^T^. LCBs in the same color are shared by all six strains, with the exception of LCBs in black which are shared by less than the six strains.

Retrieved large and small chromosomes from all the type strains were further analyzed to search synteny segments (LCBs). Figure 3 shows that the vast majority of chromosomes I and II can be pairwise aligned to its respective counterpart in *S. kushneri* AL184^T^, with 99.3–95.7% of chromosome I and 98.2–89.5% of chromosome II from *S. costicola* subsp. *costicola* LMG 11651^T^, *S. costicola* subsp. *alcaliphilus* DSM 16359^T^, *S. proteolyticus* DSM 19052^T^, *S. sharmensis* DSM 18182^T^, and *S. siamensis* JCM 14472^T^ matching a conserved region in *S. kushneri* AL184^T^. Multiple sequence alignment among the large and small chromosomes of all the six genomes under study allows identifying 87 LCBs (3,713,502 bp alignment length) for chromosome I, of which 34 LCBs (3,594,867 bp alignment length) where common to all genomes, and 30 LCBs (1,090,330 bp alignment length) for chromosome II, with 17 LCBs (1,034,054 bp alignment length) shared among all taxa (Figure 4). Although the number of common LCBs for the large chromosome was significantly smaller than the 306 common LCBs reported by Dikow and Smith (2013) for a similar comparison within the family *Vibrionaceae*, the lengths of the alignments were almost the same, which means that our common LCBs for chromosome I are fewer but longer, and actually span between 95.0 and 98.7% of the large chromosome length of the six analyzed strains.

Concerning the small chromosome, the number of common LCBs identified in our study was approximately half of those detected by Dikow and Smith (2013) for other members of the family *Vibrionaceae*, but our alignment length was more than twice longer. That means that the small chromosomes of the six *Salinivibrio* genomes were much more homologized, with percentages between 88.7 and 98.5%. These measurements were made when gaps were removed from the alignments. Therefore, in contrast to the study of Dikow and Smith (2013), no significant differences in homologization rates between large and small *Salinivibrio* chromosomes could be observed. It must be noted that the study of Dikow and Smith (2013) dealt with complete genomes, while here ordered draft genomes were employed, what might partially explain such differences.

This higher synteny in our *Salinivibrio* genomes vs. the results reported by Dikow and Smith (2013) might be due to the fact that the genomes analyzed in this study were more closely related among them (84.3% average OrthoANI and 90.0% average AAI) than the *Vibrionaceae* genomes of the mentioned study (74.1% average OrthoANI and 70.6% average AAI), thus, the higher the relatedness of the genomes under study the higher will be the synteny. To confirm this statement, we calculated the correspondence between synteny (measure as the percentage of aligned genome) and OrthoANI and AAI values among all pair of the genomes from type strains. However, the Pearson’s coefficient was only 0.21 for OrthoANI and 0.25 for AAI, indicating a poor but still significant correlation, which means that the conserved synteny of *Salinivibrio* genomes is partially due to the high average OrthoANI/AAI values among the studied genomes, but also we can hypothesize a slow speciation process or homogenization events that might be occurring in salinivibrios, a statement that needs to be tested experimentally in the future.

### Phylogenomics of the Genus *Salinivibrio*

**FIGURE 5:**
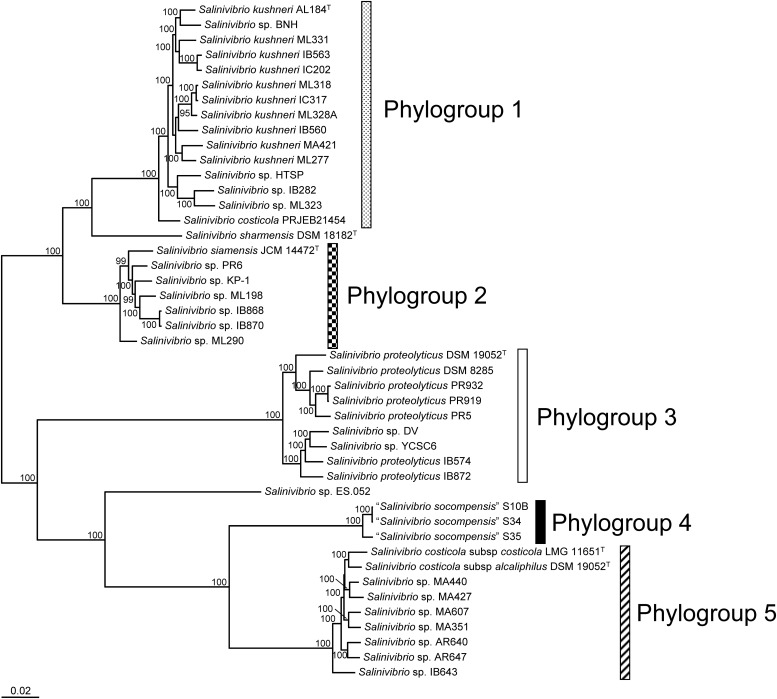
Maximum-likelihood phylogenomic tree based on the concatenation of 776 single copy core genes showing the relationships among 45 *Salinivibrio* strains whose genomes are available. Bootstrap values ≥ 70% are shown at the nodes. Bar, 0.02 nt changes per position.

Single-copy core-genome genes and proteins were employed to construct a phylogenomic tree in order to elucidate the taxonomic relationship among members of the genus *Salinivibrio*. Maximum-likelihood phylogenies based on the concatenation of 776 genes (777,643 bp alignment length) and 1,637 proteins (515,359 bp alignment length) yielded two very similar trees with high bootstrap support, where five different phylogroups and two phylotypes can be distinguished (Figure 5 and Supplementary Figure 2). Phylogroup 1 corresponds to *S. kushneri* strains, and includes strains previously affiliated to this species (López-Hermoso et al., 2018b) as well as three other strains originally named as *Salinivibrio* sp. and another probably mislabeled strain initially designated as *S. costicola*. Phylogroup 2 is formed by *S. siamensis* JCM 14472^T^ and other five strains identified as members of this species in a previous MLSA approach (López-Hermoso et al., 2017b) as well as one additional strain labeled as *Salinivibrio* sp. KP-1. Phylogroup 3 agrees on the monophyletic group defined by López-Hermoso et al. (2018a) to emend the description of *S. proteolyticus* but it also groups two additional strains not analyzed by those authors, which we prove to belong to the aforementioned species. Phylogroup 4 consists of the three strains proposed by Gorriti et al. (2014) as a new *Salinivibrio* species with the not-yet validated name “*S. socompensis.*” Finally, phylogroup 5 exactly fits the cluster defined by López-Hermoso et al. (2017b) formed by nine strains, including the two subspecies of *S. costicola*, *S. costicola* subsp. *costicola* and *S. costicola* subsp. *alcaliphilus*. Actually, phylogenomically, the two subspecies cannot be clearly differentiated (especially when the tree is constructed using the concatenated core genes), a maybe the *Salinivibrio* subspecies rank should be revised. Furthermore, two *Salinivibrio* strains could neither be included in any of the above phylogroups, nor formed a phylogroup themselves, so they were defined as phylotypes. One of them consists of the type strain of *S. sharmensis*, DSM 18182^T^, and the other is an unnamed *Salinivibrio* strain, ES.052, isolated from an intertidal microbial mat in Elkhorn Slough (California), which probably constitutes a new species of *Salinivibrio* not described yet.

OrthoANI values calculated for all-vs.-all pairs (Supplementary Figure 3) confirmed that the aforementioned phylogroups and phylotypes are actually different species of the genus *Salinivibrio*. The OrthoANI values within each phylogroup were always above 95%, whereas, the values among phylogroups/phylotypes were in all cases far below 95%, the threshold value proposed for species boundaries (Richter and Rosselló-Móra, 2009), thus supporting our proposal to designate each phylogroup and phylotype to a different *Salinivibrio* species. We also propose to rename the *Salinivibrio* strains used here as follows: all strains belonging to phylogroup 1 should be labeled as *S. kushneri*, those of phylogroup 2 as *Salinivibrio siamensis*, and those of phylogroup 3 as *S. proteolyticus*. Strains of phylogroup 5 should be relabeled as *S. costicola*, without indicate the subspecies to which each strain is affiliated, with the exception of the type strains of the subspecies. Finally, phylogroup 4 and the two phylotypes will retain their actual designation (Table 1). For some genome pairs (i.e., strains of phylogroup 3 vs. strains of phylogroups 4 and 5) the OrthoANI values were slightly below 80%, and so, those genomes are too divergent to use nucleotide level comparisons. In consequence, AAI values were estimated for all-vs.-all genome pairs (Supplementary Figure 3) confirming that phylogroups 3, 4, and 5, as well as the phylotype *Salinivibrio* sp. ES.052, constitute separate species. However, AAI results equal or above 95% among strains of phylogroups 1 and 2, and for the phylotype *S. sharmensis* DSM 18182^T^, suggest that they all might form a single species. Nevertheless, these strains share more than 90% ANI in all cases, therefore, according to Rodriguez-R and Konstantinidis (2014) for such closely related strains ANI offers a robust resolution and should be used instead. For that reason, we suggest maintaining our proposal of phylogroups 1 and 2, and phylotype *S. sharmensis* DSM 18182^T^ as independent species within this genus.

Orthologous gene (OGs) cluster analysis based on amino acid and nucleotide sequences was performed to define the pan-genome of the genus *Salinivibrio*. By using translated amino acid sequence comparison of the 45 analyzed genomes, the pan-genome is composed of 5,570 OGs, of which 2,080 OGs are common to all taxa (core-genome), and 3,490 OGs constitute the variable-genome (Table 2). If the nucleotide sequences are used, then pan-genome is composed of larger OGs, 7,462, but the core-genome decreases up to 1,211 OGs while the accessory-genome rises up to 6,251 OGs (Table 2). The smaller pan-genome when using protein sequences was expected due to the fact that a lower cut-off value (40% vs. 70% sequence identity) was set for the clustering. However, given that protein sequences are more conservative than nucleotide sequences, it is not surprisingly the bigger core-genome obtained when analyzing translated amino acid sequences, especially if the genomes under study have diverged too much (OrthoANI values < 80%). A similar study within the family *Vibrionaceae* detected 6,629 OGs of which 1,882 OGs where found in all 11 proteomes under study (Lilburn et al., 2009), but this smaller core-genome is probably attributable to the fact that the analysis was performed on genomes belonging to different genera. A more recent research only focused in a single genus, *Vibrio*, and conducted with 20 proteomes (corresponding to 20 different species) yielded a large pan-genome of 21,844 OGs, with only 1,630 OGs common to all taxa (Lin et al., 2018), which may be explained by the lower genomic relatedness (measured by OrthoANI/AAI values) among the *Vibrio* genomes. Therefore, members of the genus *Salinivibrio* appear to have higher genetic relatedness within the group than strains of the genus *Vibrio*. These findings are consistent with our hypothesis that there should be an homogenizing force acting on the salinivibrios, what could explain the few number of species described so far, a puzzling occurrence for a genus so often isolated from hypersaline environments all over the world (Herzog et al., 2016; Ashengroph, 2017; Fernández-Delgado et al., 2017; López-Hermoso et al., 2017b; Selvarajan et al., 2017; Le and Yang, 2018; Arias et al., 2019). However, this hypothesis awaits experimental testing in the future.

**TABLE 2:** Pan- and core-genome features of the *Salinivibrio* genomes based on translated protein and nucleotide gene sequences.

**Characteristic**	**Protein-based pan- and core-genomes**	**Nucleotide-based pan- and core-genomes**
Genome #	45	45
Pan-genome (OGs)	5,570	7,462
Core-genome (OGs)	2,080	1,211
Core-genome 90% (OGs)	2,430	1,356
Core-genome 80% (OGs)	2,501	1,660
Variable-genome (OGs)	3,490	6,251
Core-/pan-genome (%)	37.3	16.2
Mean OGs per genome	3,007.8	2,986.5
Mean OGs/pan-genome (%)	54.0	40.0
Core-genome/mean OGs (%)	69.2	40.5

**FIGURE 6:**
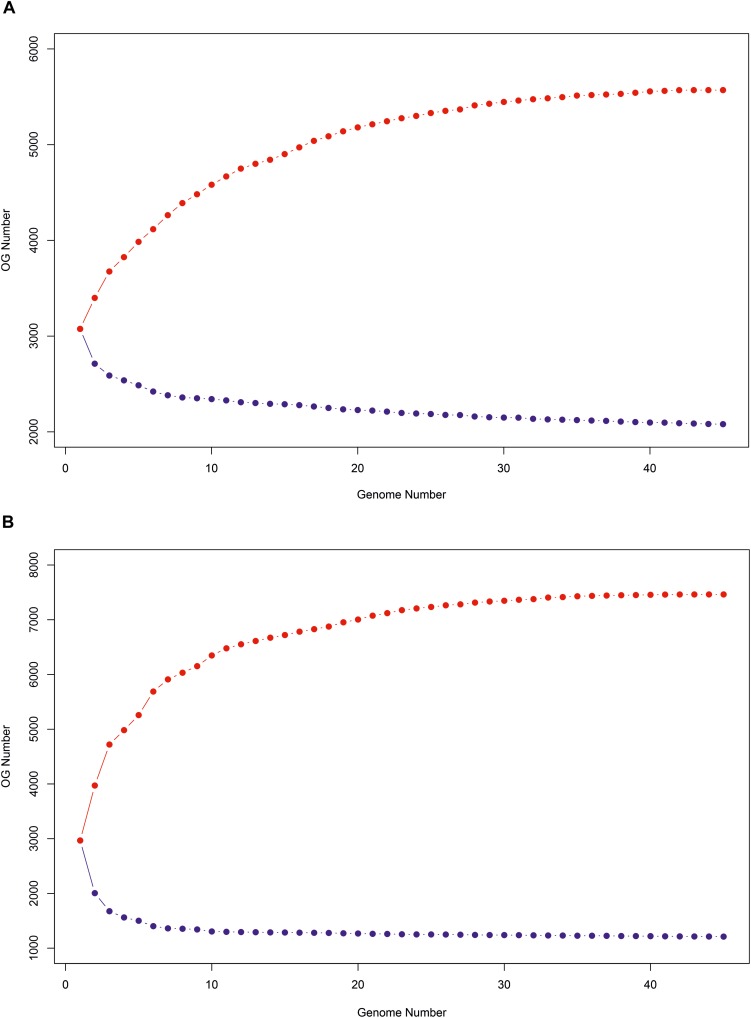
Progression of the pan-genome (red) and core-genome (blue) of the 45 *Salinivibrio* genomes based on protein **(A)** and gene **(B)** sequences.

The progression of the pan- and core-genomes after random samplings was calculated (Figure 6). As can be observed, pan-genome based on gene sequences gradually increased when more genomes were considered up to reach 30 genomes, and after that it would remain relatively constant, even as many more genomes were added. On the contrary, the nucleotide-based core-genome rapidly decreased when more genomes were added, becoming relatively stable after 7 genomes were considered. Similarly, the pan- and core-genomes based on protein sequences followed the same tendency, but the saturation of the curves occurred with a higher number of genomes, i.e., 40 and 38, respectively, because protein search is more sensitive.

### Distribution and *Salinivibrio* Species Diversity Based on Culture-Independent Approaches

To provide cultivation-independent assessment of the *Salinivibrio* species diversity, the spatial distribution of the genus was analyzed based on the origin of 16S rRNA gene sequences found in SILVA database (release 132), as well as the source of the 45 completely sequenced strains analyzed in this study. As can be observed (Supplementary Figure 4), the genus *Salinivibrio* possess a high dispersal potential, with a cosmopolitan distribution. Besides, strains/sequences belonging to different species have been isolated from the same region, which means that different species might have similar habitat preferences.

Although isolation of new *Salinivibrio* species has not been often achieved, new culture-independent techniques based on high throughput sequencing can provide some clues about the species diversity in the natural environment. To this end, we analyzed several 16S rRNA gene amplicon datasets from recent studies (i.e., Tkavc et al., 2011; Zhang et al., 2016, 2017; Crisler et al., 2019) reporting presence of salinivibrios for OTUs relative abundance and taxonomic assignment. Considering 97% as the species cut-off value, we were able to identify 11, 118, 135, and zero different OTUs in the datasets of Tkavc et al. (2011), Zhang et al. (2016, 2017), and Crisler et al., 2019, respectively. Furthermore, we downloaded all 16S rRNA gene sequences from SILVA Nr99 database (release 132) labeled as *Salinivibrio* and performed the clustering analysis with 97% sequence identity, obtaining 48 different OTUs which could represent 48 putative different species of *Salinivibrio*. Although these culture-independent approaches might indicate, in some cases, a broader species diversity than that expected based on culture techniques it must be noticed that amplicon metagenomic datasets contained partial 16S rRNA gene sequences (∼200–691 bp average length) and, therefore, OTUs clustering might be biased and inflate natural diversity. In any case, our results show that, although genomic conservation within salinivibrios is higher compared to other members of the family *Vibrionaceae* (based on synteny and core/pangenome analyses), new species might exist in the studied habitats and, therefore, additional attempts to isolate and to describe them should be performed.

To provide a whole-genome view in the environments where salinivibrios may be theoretically more abundant, we performed fragment recruitment analyses using several shotgun metagenomic datasets (Supplementary Table 1) against all the type strains of the genus. Salinivibrios showed recruitment in saline water and soil metagenomes, with lower recruitment values at hypersaline metagenomes (Supplementary Figure 5). Surprisingly, the highest values were not obtained at intermediate salinities where member of this genus are frequently isolated, but at lower salinities, being especially abundant in an Iranian saline lake with 5% NaCl. Therefore, these recruitment plots suggest that future efforts to search for new *Salinivibrio* species might be conducted sampling lower salinity environments.

### Genomic Attributes of the Genus *Salinivibrio*

Annotation and comparison of the 44 *Salinivibrio* genomes considered in this study indicated that the ten most representative subsystems included genes related to flagellar synthesis and regulation, tRNA, large and small ribosomal subunits, RNA methylation, methionine synthesis, cytoskeleton, serine-glyoxylate cycle, DNA repair, and phosphate metabolism (Supplementary Figure 6).

A total of 46 genes related to synthesis and regulation of flagellum were detected in the studied *Salinivibrio* genomes, which might be expected since salinivibrios are motile bacteria by means of one pollar flagellum (Mellado et al., 1996). Among them, *fliDC* (flagellar filament regulator), *flgLK* (filament-hook joint), and *flgEKD* (flagellar hook regulator) genes were detected. Additionally, *motAB* genes related to transport between flagellum and extracellular fluid were identified (Kuchma et al., 2015). Previous studies have confirmed the fast evolutionary rate of these regulators (Soutourina and Bertin, 2003; McCarter, 2006). Although the flagellar regulation genes have not been completely elucidated, each bacterial species usually possesses a different regulation network. However, the salinivibrios under study present the same flagellar regulation network, which indicate the high homogeneity among this bacterial genus.

Salinivibrios are described as facultatively anaerobic bacteria, so, we looked for genes involved in the anaerobic respiration. Two kinds of reductases were detected in all the studied genomes, AsrR (arsenate reductase) and FIR (ferredoxin-NADPH reductase). Arsenate, in despite of its toxicity, can be used by some microorganisms as an electron acceptor in the anaerobic respiration (Saltikov and Newman, 2003; Ruebush et al., 2006), what lead us to hypothesize that members of the genus *Salinivibrio* might metabolize arsenate under anaerobic conditions. Futhermore, a cytochrome cbb (3) oxidase complex implicated in microaerobic respiration (Pitcher and Watmough, 2004) was identified. This complex might provide a better adaptation to microaerobic environments, pointing to evolutionary modifications of salinivibrios to thrive at low oxygen concentrations, such as those present in hypersaline aquatic habitats (Rodríguez-Valera, 1993; Ventosa, 2006).

Osmotic response of *Salinivibrio* strains is mediated by accumulation of cytoplasmatic compatible solutes (also know as the “salt-out” strategy) which can be synthesized *de novo* or captured from the environment (Zhu et al., 2008, 2010). Ectoine is probably the key compatible solute in osmotic adaptation of salinivibrios (Zhu et al., 2008). The complete cluster of genes involved in ectoine synthesis, *ectABC*, was present in all the studied genomes, but *ectD* gene, responsible of ectoine to hydroxyectoine conversion was missed in some genomes. Ectoine transportation to cellular inner is accomplished by a TeaABC transporter (Grammann et al., 2002), which could not be detected in the *Salinivibrio* genomes, therefore, we deducted that salinivibrios use ectoine to balance osmotic stress by *de novo* synthesis instead of by transportation. Glycine betaine is another compatible solute widely used by bacteria that is synthesized from choline (involving *betAB* genes) or choline O-sulfate (involving *betABC* genes) (Lidbury et al., 2015). All the studied genomes codified the *betA* and *betB* genes, but *betC* was absent, so salinivibrios can only utilize choline, but not choline O-sulfate. As expected, the gene for a high-affinity choline uptake protein (*betT*) was codified in all the genomes. The glycine betaine transporter (*opuD*) was only present in *Salinivibrio* strains belonging to phylogroups 1 and 4, being synthesized and not transported in the other strains. Trehalose can be used by bacteria as a compatible solute in response to osmotic and thermal stresses (Avonce et al., 2006). *Salinivibrio* genomes contained the gene cluster *otsAB*, required for trehalose synthesis, but they lack the enzymes for its degradation, what means that trehalose is not used as a carbon and energy source.

Concerning the nitrogen metabolism, genes related to nitrogen fixation, nitrification, denitrification, and assimilatory nitrate reduction were not identified within salinivibrios, in agreement to the results of Gorriti et al. (2014). Ammonium assimilation is carried out either by the GDH (glutamate dehydrogenase) or by the GS/GOGAT (glutamine synthetase/glutamate synthase) pathways. Given that salinivibrios do not possess the enzymatic activity to reduce nitrate, the ammonium uptake that will be incorporated into carbon skeletons is achieved by mean of an Amt transporter.

Although a total of 14 pathways and 44 different enzymes are currently known leading to PHA (poly-hydroxyalkanoate) synthesis (Meng et al., 2015), PHA synthase (PhaC) plays a key role in the PHA biosynthetic pathway (Chek et al., 2017). In the *Salinivibrio* genomes under study, only *phaC* gene was detected, but none of the other PHA synthesis-related genes (including the PHA depolymerase). Therefore, it is possible that salinivibrios might accumulate PHA, but they cannot perform PHA degradation.

## Conclusion

We have sequenced the complete genome of the type strain of the species *S. kushneri* AL184^T^, which, unsurprisingly, is constituted by two chromosomes, as usual for other members of the family *Vibrionaceae*. This is the first closed genomic sequence currently available within this genus. We have corroborated that PacBio reads with above 200× average per-base coverage are enough to recover a high quality complete bacterial genome. The complete genome is very useful to identify rearrangements and to order contigs of closely related draft genomes. Synteny analysis among the genomes of the type strains of the genus *Salinivibrio* has demonstrated the high degree of homologization within this genus. This might evidence a slower evolutionary rate in salinivibrios, what would explain the surprising few numbers of species validly described so far given how often new *Salinivibrio* strains are isolated from different environments, but this hypothesis should be further tested experimentally. Nevertheless, metagenomic analyses suggest that a broader species diversity might exist in natural environments, although these results may be regarded with caution, and that a higher abundance of salinivibrios probably occurs at lower salinity concentrations.

Currently, the genus *Salinivibrio* includes five species with validly described names, as well as a non-yet-validated species name. Our phylogenomic analysis supports the taxonomic status of those six *Salinivibrio* species and, moreover, evidence the existence of an additional species represented by the strain *Salinivibrio* sp. ES.052. Besides, the taxonomic distinction between the two subspecies of *S. costicola* is not clear according to our results and, moreover, there are no weighted phenotyphic differences between both subspecies beyond optimal pH supporting growth and some biochemical features which may also differ among strains of the same species (Romano et al., 2005) so, perhaps, the subspecies status of this species should be deeply revised.

Genomic features of the 45 studied strains also agreed with the high homogeneity of this genus. Phenotypic characteristics described for the member of this group concur with the information derived from the annotated genomes. The only exception is the reduction of nitrate and nitrite, which has been observed in laboratory experiments (Romano et al., 2005, 2011; Chamroensaksri et al., 2009; López-Hermoso et al., 2018a, b), but could not be detected in the analyzed genomes. Another interesting feature detected in the complete genome of *S. kusnheri* AL184^T^ is the large number of rRNA genes (nine complete ribosomal clusters), which is expected for bacteria with a fast growing rate in artificial media.

## Data Availability

The large and small chromosomes of *Salinivibrio kushneri* AL184^T^ generated for this study can be found in the GenBank under the accession numbers CP040021 and CP040022, respectively.

## Author Contributions

AV, RH, and CS-P conceived and designed the study. CL-H and CS-P performed the laboratory experiments. RH, CL-H, CS-P, KK, and AV analyzed and interpreted the data, and discussed and critically revised the manuscript. RH and AV drafted the manuscript. All authors read and approved the final manuscript.

## Conflict of Interest Statement

The authors declare that the research was conducted in the absence of any commercial or financial relationships that could be construed as a potential conflict of interest.
