# Supplementary material for: Comparative Genomics and Phylogenomic Analysis of the Genus Salinivibrio
Source: Front Microbiol. 2019 Sep 11;10:2104. doi: 10.3389/fmicb.2019.02104 (PMC6749099; doi:10.3389/fmicb.2019.02104)
Supplement: Supplementary file 1 [file Data_Sheet_1.doc]

***Supplementary Material***

**Comparative genomics and phylogenomic analysis of the genus *Salinivibrio***

**Rafael R. de la Haba^1^, Clara López-Hermoso^1^, Cristina Sánchez-Porro^1^, Konstantinos T. Konstantinidis^2^, Antonio Ventosa^1*^**

^1^Department of Microbiology and Parasitology, Faculty of Pharmacy, University of Sevilla, Sevilla, Spain

^2^School of Civil and Environmental Engineering, Georgia Institute of Technology, Atlanta, Georgia, USA

**^*^ Correspondence:**

Dr. Antonio Ventosa

[ventosa@us.es](mailto:ventosa@us.es)

**Supplementary Figures**


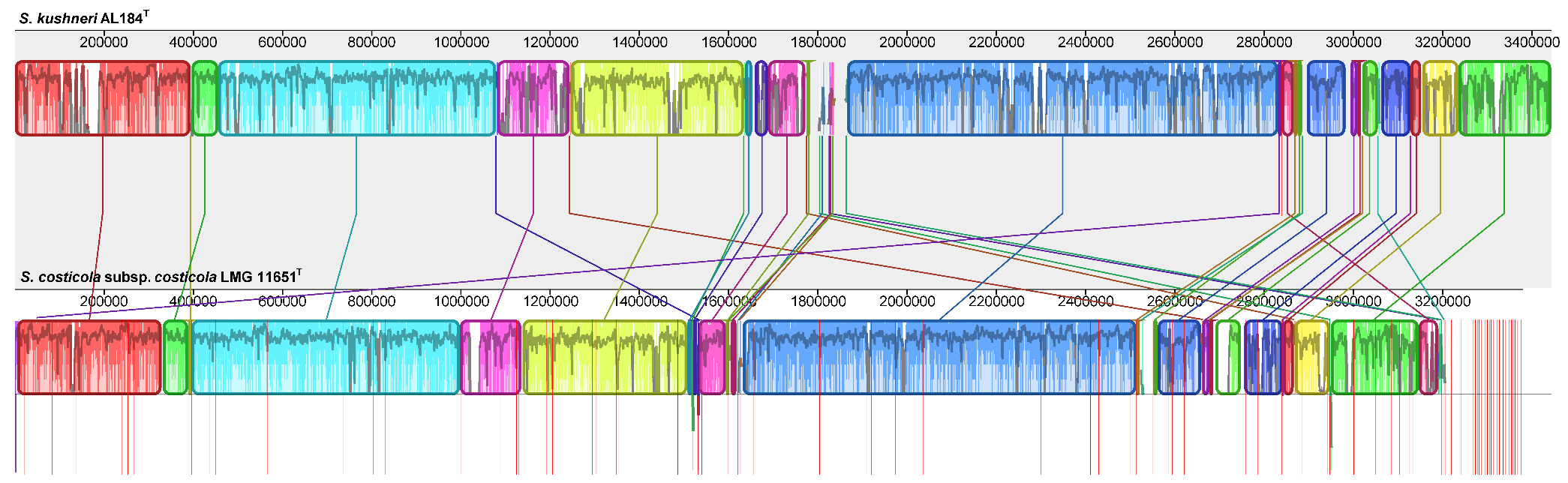
**A**


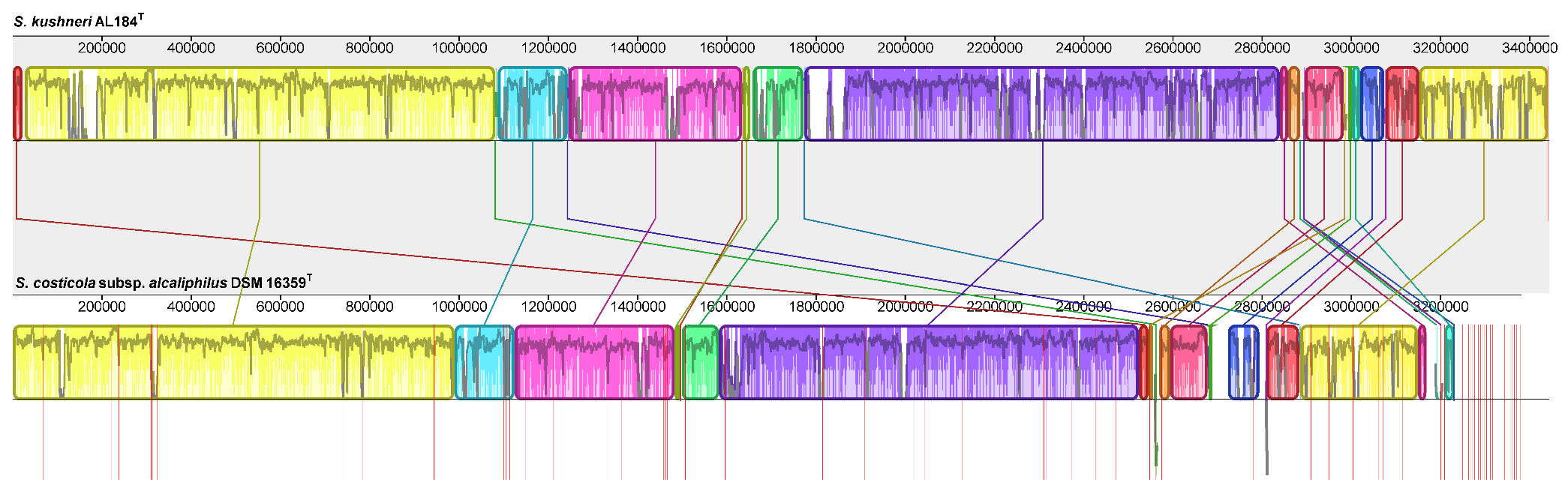
**B**

**C**

**
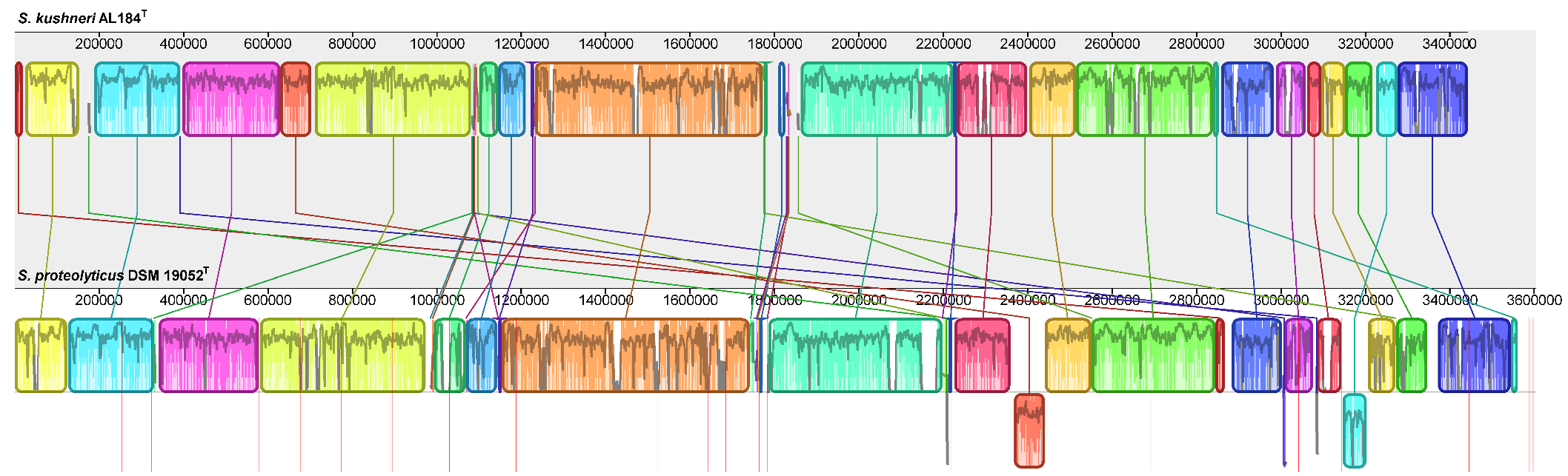
**

**D**

**
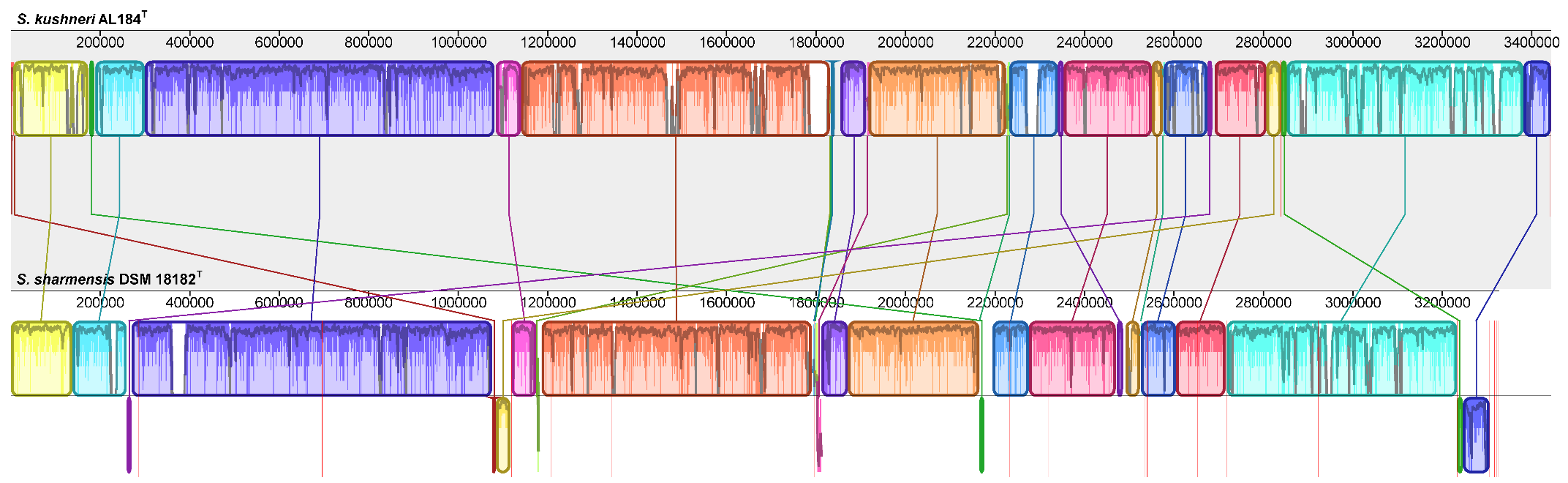
**


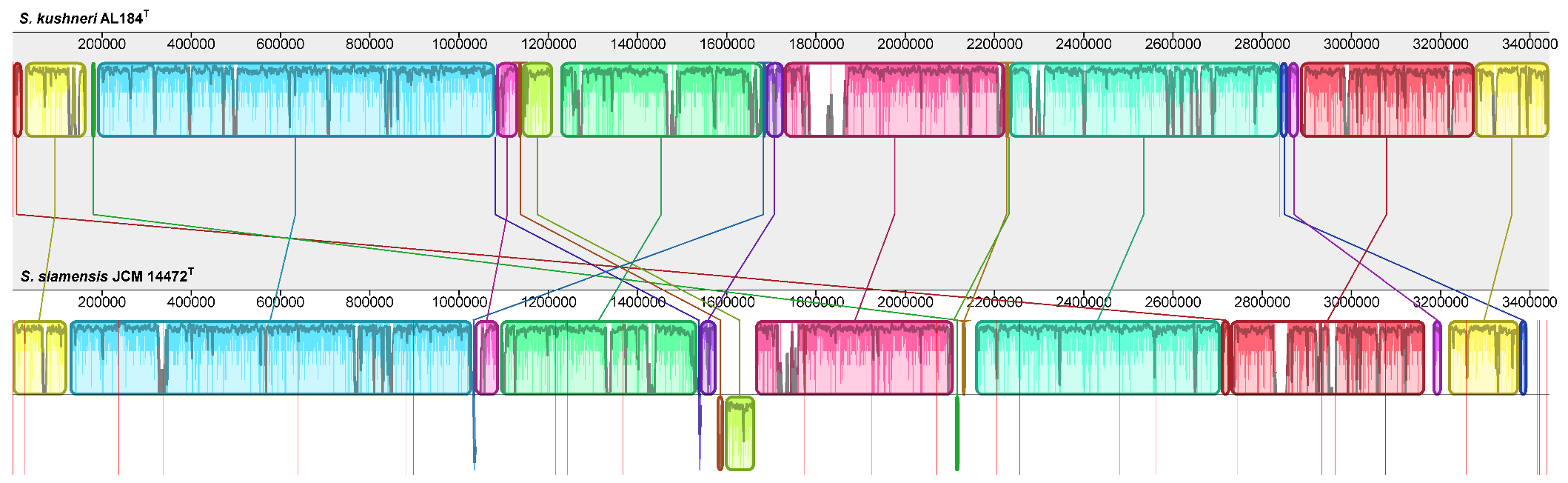
**E**

**Supplementary Figure 1. *Salinivibrio* draft genome ordering by mean of pairwise alignment between *S. kushneri* AL184^T^ and *S. costicola* subsp. *costicola* LMG 11651^T^ (A), *S. costicola* subsp. *alcaliphilus* DSM 16359^T^ (B), *S. proteolyticus* DSM 19052^T^ (C), *S. sharmensis* DSM 18182^T^ (D), and *S. siamensis* JCM 14472^T^ (E).**

**Supplementary Figure 2. Maximum-likelihood phylogenomic tree based on the concatenation of 1,637 single copy core proteins showing the relationships among 45 *Salinivibrio* strains whose genomes are available.** Bootstrap values are shown at the nodes. Bar, 0.02 nt changes per position.

**Supplementary Figure 3. OrthoANI (lower triangle) and AAI (upper triangle) values among the genomes of the 45 strains of the genus *Salinivibrio* under study.**


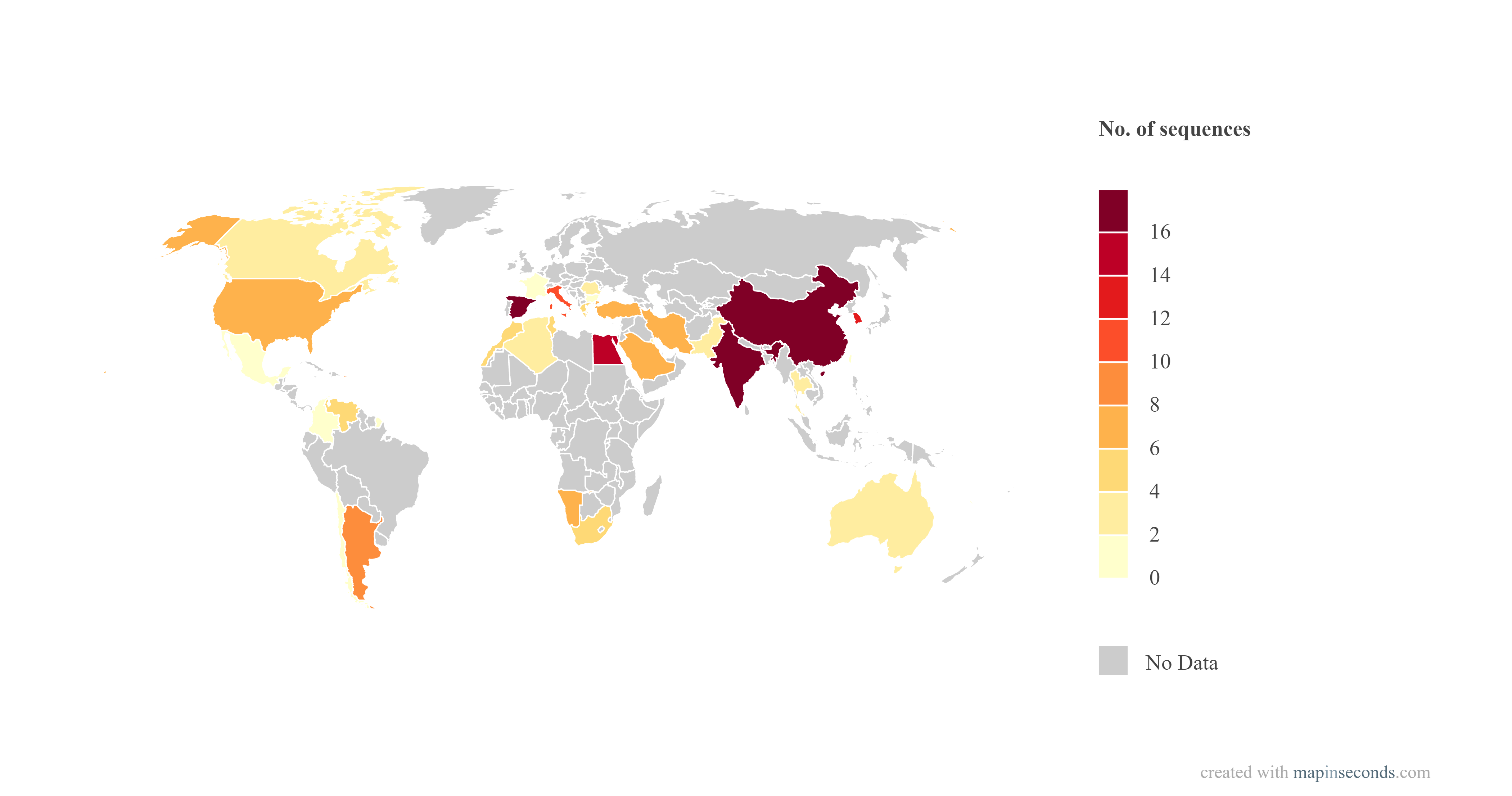
**Supplementary Figure 4. Worldwide distribution of the genus *Salinivibrio*.** Source countries were *Salinivibrio* 16S rRNA gene sequences present in SILVA database and *Salinivibrio* genome sequences from GenBank were detected are colored according to abundancy.

**Supplementary Figure 5. Recruitment plots of type strains of species of *Salinivibrio* against different metagenomic dataset.** In each panel the Y axis represents the identity percentage and X axis represents the genome length. A restrictive cut-off 95 % of nucleotide identity in at least 30 bp of the metagenomic read was used. The black dashed line shows the threshold for presence of same species (95% identity). For metagenomic dataset abbreviations see Supplementary Table 3.


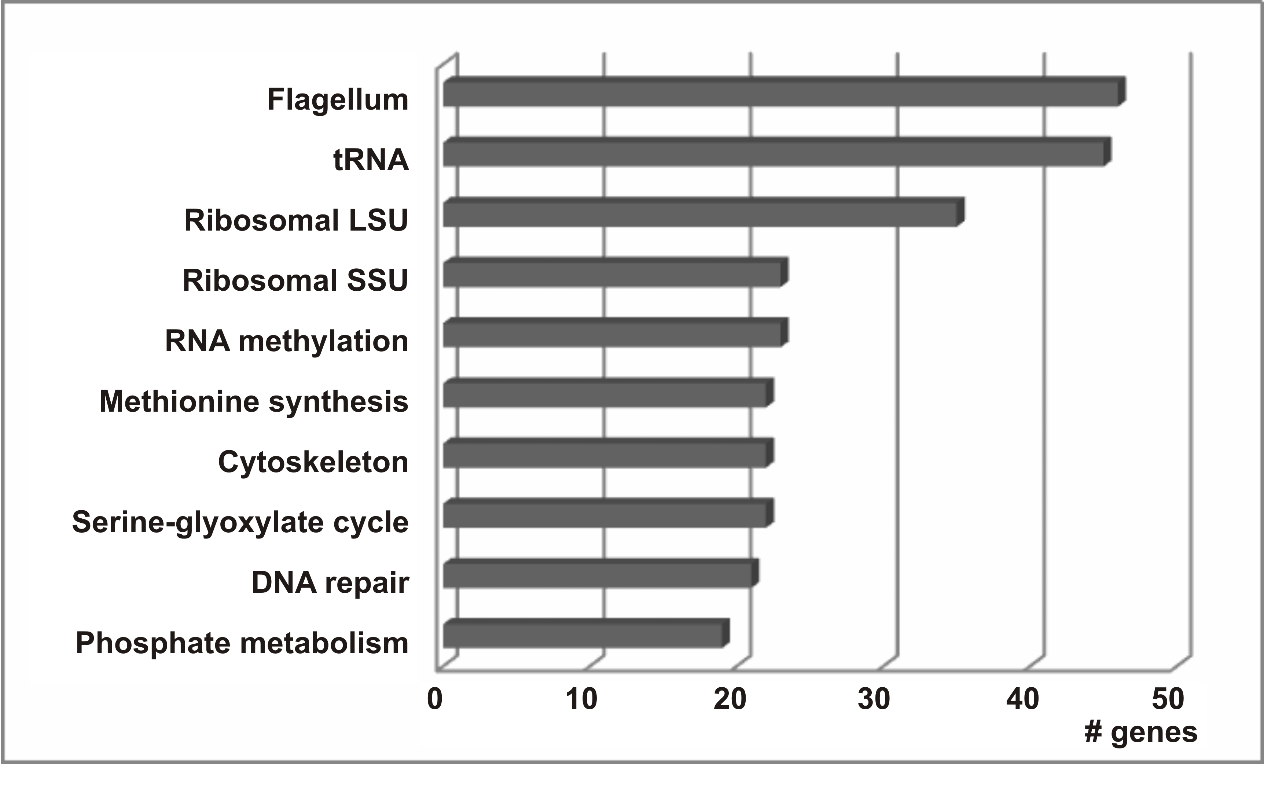
**Supplementary Figure 6. Bar plot of the ten most abundant subsystems present in *Salinivibrio* genomes showing the number of genes included within each subsystem.**

**Supplementary Tables**

**Supplementary Table 1. Features of the different shotgun metagenomic databases from hypersaline habitats (water and soils) utilized in this study.**

| **Database Name** | **Habitat** | **Salinity** | **Accesion number** | **Reference** |
| --- | --- | --- | --- | --- |
| Meyghan5 | Hypersaline lake | 5 % NaCl | ERS1455389 | Naghoni et al., 2017 |
| SS13 | Saltern | 13 % NaCl | SRX328504 | Fernández *et al.*, 2014a |
| Meyghan18 | Hypersaline lake | 18 % NaCl | ERS1455390 | Naghoni et al., 2017 |
| SS19 | Saltern | 19 % NaCl | SRX090228 | Ghai *et al.*, 2011 |
| IC21 | Saltern | 21 % NaCl | SRX352042 | Fernández *et al.*, 2014b |
| Tyrrell 0.1 | Saltern | 29 % NaCl | SRR5637210 | Podell *et al.*, 2014 |
| Tyrrell 0.8 | Saltern | 29 % NaCl | SRR5637211 | Podell *et al.*, 2014 |
| Meyghan30 | Hypersaline lake | 30 % NaCl | ERS1455391 | Naghoni et al., 2017 |
| S7 | Saltern | 30 % NaCl | SRR8921445 | Unpublished |
| SS33 | Saltern | 33 % NaCl | SRX347883 | Fernández *et al.*, 2014a |
| SS37 | Saltern | 37 % NaCl | SRX090229 | Ghai *et al.*, 2011 |
| Cahuill | Saltern | 34 % NaCl | SRX680116 | Plominsky *et al.*, 2014 |
| SMO1 | Saline soil | 24.0 dS / m | SRR5753725 | Vera-Gargallo *et al.*, 2018 |
| SMO2 | Saline soil | 54.4 dS / m | SRR5753724 | Vera-Gargallo *et al.*, 2018 |
| Gujarat | Saline soil | ND | ERP005612 | Patel *et al.*, 2015 |

**Supplementary Table 2. Proposed contig ordering and tentative assignation to chromosome I or II for the draft genome sequences of the type strains of the species and subspecies of the genus *Salinivibrio*.** Ordered GenBank contig accession numbers are indicated followed by their orientation (+, forward; -, reverse complement).

| **Strain** | **Ordered contigs assigned to chromosome 1 (orientation)** | **Ordered contigs assigned to chromosome II (orientation)** | **Unassigned contigs** |
| --- | --- | --- | --- |
| *S. costicola* subsp. *costicola* LMG 11651^T^ | NZ_AQOF01000033.1 (+), NZ_AQOF01000111.1 (+), NZ_AQOF01000043.1 (-), NZ_AQOF01000016.1 (+), NZ_AQOF01000013.1 (+), NZ_AQOF01000004.1 (+), NZ_AQOF01000082.1 (-), NZ_AQOF01000091.1 (+), NZ_AQOF01000090.1 (+), NZ_AQOF01000131.1 (+), NZ_AQOF01000005.1 (+), NZ_AQOF01000105.1 (+), NZ_AQOF01000132.1 (+), NZ_AQOF01000007.1 (+), NZ_AQOF01000116.1 (-), NZ_AQOF01000092.1 (+), NZ_AQOF01000001.1 (-), NZ_AQOF01000026.1 (-), NZ_AQOF01000010.1 (+), NZ_AQOF01000037.1 (-), NZ_AQOF01000023.1 (+), NZ_AQOF01000067.1 (+), NZ_AQOF01000062.1 (-), NZ_AQOF01000083.1 (+), NZ_AQOF01000031.1 (-), NZ_AQOF01000006.1 (+), NZ_AQOF01000059.1 (+), NZ_AQOF01000149.1 (-), NZ_AQOF01000094.1 (-), NZ_AQOF01000056.1 (-), NZ_AQOF01000144.1 (-), NZ_AQOF01000054.1 (-), NZ_AQOF01000113.1 (-), NZ_AQOF01000060.1 (+), NZ_AQOF01000120.1 (-), NZ_AQOF01000014.1 (-), NZ_AQOF01000119.1 (+), NZ_AQOF01000055.1 (-), NZ_AQOF01000136.1 (-), NZ_AQOF01000042.1 (+), NZ_AQOF01000115.1 (+), NZ_AQOF01000009.1 (-), NZ_AQOF01000088.1 (+), NZ_AQOF01000078.1 (+), NZ_AQOF01000075.1 (-), NZ_AQOF01000112.1 (+), NZ_AQOF01000025.1 (-), NZ_AQOF01000081.1 (+), NZ_AQOF01000137.1 (-), NZ_AQOF01000046.1 (+), NZ_AQOF01000123.1 (+), NZ_AQOF01000141.1 (-), NZ_AQOF01000065.1 (+), NZ_AQOF01000117.1 (+), NZ_AQOF01000099.1 (+), NZ_AQOF01000103.1 (+), NZ_AQOF01000057.1 (-), NZ_AQOF01000051.1 (-), NZ_AQOF01000048.1 (+), NZ_AQOF01000019.1 (-), NZ_AQOF01000071.1 (-), NZ_AQOF01000003.1 (+), NZ_AQOF01000077.1 (-), NZ_AQOF01000096.1 (+), NZ_AQOF01000086.1 (+), NZ_AQOF01000041.1 (+), NZ_AQOF01000045.1 (+), NZ_AQOF01000163.1 (+), NZ_AQOF01000164.1 (+), NZ_AQOF01000089.1 (-), NZ_AQOF01000017.1 (-), NZ_AQOF01000140.1 (-), NZ_AQOF01000029.1 (-), NZ_AQOF01000018.1 (-), NZ_AQOF01000027.1 (-), NZ_AQOF01000064.1 (-), NZ_AQOF01000015.1 (+), NZ_AQOF01000030.1 (-), NZ_AQOF01000061.1 (+), NZ_AQOF01000053.1 (+), NZ_AQOF01000008.1 (+), NZ_AQOF01000022.1 (+), NZ_AQOF01000063.1 (+), NZ_AQOF01000034.1 (-), NZ_AQOF01000085.1 (+), NZ_AQOF01000098.1 (+), NZ_AQOF01000032.1 (-), NZ_AQOF01000102.1 (-), NZ_AQOF01000028.1 (+), NZ_AQOF01000066.1 (-) | NZ_AQOF01000036.1 (-), NZ_AQOF01000069.1 (+), NZ_AQOF01000084.1 (+), NZ_AQOF01000110.1 (+), NZ_AQOF01000138.1 (-), NZ_AQOF01000040.1 (-), NZ_AQOF01000150.1 (+), NZ_AQOF01000146.1 (+), NZ_AQOF01000039.1 (-), NZ_AQOF01000047.1 (+), NZ_AQOF01000020.1 (-), NZ_AQOF01000058.1 (+), NZ_AQOF01000121.1 (+), NZ_AQOF01000052.1 (-), NZ_AQOF01000125.1 (-), NZ_AQOF01000002.1 (+), NZ_AQOF01000038.1 (-), NZ_AQOF01000011.1 (+), NZ_AQOF01000101.1 (-), NZ_AQOF01000114.1 (+), NZ_AQOF01000024.1 (-), NZ_AQOF01000079.1 (+), NZ_AQOF01000021.1 (-), NZ_AQOF01000072.1 (-), NZ_AQOF01000068.1 (-), NZ_AQOF01000076.1 (-), NZ_AQOF01000012.1 (-), NZ_AQOF01000095.1 (-), NZ_AQOF01000106.1 (+), NZ_AQOF01000080.1 (+), NZ_AQOF01000044.1 (+), NZ_AQOF01000104.1 (-), NZ_AQOF01000087.1 (-), NZ_AQOF01000074.1 (+), NZ_AQOF01000109.1 (+), NZ_AQOF01000126.1 (+), NZ_AQOF01000050.1 (+), NZ_AQOF01000073.1 (-), NZ_AQOF01000093.1 (-) | NZ_AQOF01000134.1, NZ_AQOF01000035.1, NZ_AQOF01000049.1, NZ_AQOF01000070.1, NZ_AQOF01000097.1, NZ_AQOF01000100.1, NZ_AQOF01000107.1, NZ_AQOF01000108.1, NZ_AQOF01000118.1, NZ_AQOF01000122.1, NZ_AQOF01000124.1, NZ_AQOF01000127.1, NZ_AQOF01000128.1, NZ_AQOF01000129.1, NZ_AQOF01000130.1, NZ_AQOF01000133.1, NZ_AQOF01000135.1, NZ_AQOF01000139.1, NZ_AQOF01000142.1, NZ_AQOF01000143.1, NZ_AQOF01000145.1, NZ_AQOF01000147.1, NZ_AQOF01000148.1, NZ_AQOF01000151.1, NZ_AQOF01000152.1, NZ_AQOF01000153.1, NZ_AQOF01000154.1, NZ_AQOF01000155.1, NZ_AQOF01000156.1, NZ_AQOF01000157.1, NZ_AQOF01000158.1, NZ_AQOF01000159.1, NZ_AQOF01000160.1, NZ_AQOF01000161.1, NZ_AQOF01000162.1, NZ_AQOF01000165.1, NZ_AQOF01000166.1, NZ_AQOF01000167.1, NZ_AQOF01000168.1, NZ_AQOF01000169.1, NZ_AQOF01000170.1, NZ_AQOF01000171.1, NZ_AQOF01000172.1, NZ_AQOF01000173.1, NZ_AQOF01000174.1, NZ_AQOF01000175.1, NZ_AQOF01000176.1, NZ_AQOF01000177.1, NZ_AQOF01000178.1, NZ_AQOF01000179.1, NZ_AQOF01000180.1, NZ_AQOF01000181.1, NZ_AQOF01000182.1, NZ_AQOF01000183.1, NZ_AQOF01000184.1, NZ_AQOF01000185.1, NZ_AQOF01000186.1, NZ_AQOF01000187.1, NZ_AQOF01000188.1, NZ_AQOF01000189.1, NZ_AQOF01000190.1, NZ_AQOF01000191.1, NZ_AQOF01000192.1, NZ_AQOF01000193.1, NZ_AQOF01000194.1, NZ_AQOF01000195.1, NZ_AQOF01000196.1, NZ_AQOF01000197.1, NZ_AQOF01000198.1, NZ_AQOF01000199.1, NZ_AQOF01000200.1, NZ_AQOF01000201.1, NZ_AQOF01000202.1 |
| *S. costicola* subsp. *alcaliphilus* DSM 16359^T^ | NZ_MUFR01000025.1 (+), NZ_MUFR01000063.1 (-), NZ_MUFR01000060.1 (-), NZ_MUFR01000073.1 (+), NZ_MUFR01000002.1 (+), NZ_MUFR01000051.1 (-), NZ_MUFR01000016.1 (-), NZ_MUFR01000082.1 (-), NZ_MUFR01000059.1 (-), NZ_MUFR01000007.1 (-), NZ_MUFR01000012.1 (-), NZ_MUFR01000040.1 (-), NZ_MUFR01000015.1 (+), NZ_MUFR01000004.1 (+), NZ_MUFR01000030.1 (-), NZ_MUFR01000070.1 (-), NZ_MUFR01000001.1 (-), NZ_MUFR01000028.1 (-), NZ_MUFR01000043.1 (+), NZ_MUFR01000013.1 (+), NZ_MUFR01000092.1 (+), NZ_MUFR01000080.1 (-), NZ_MUFR01000076.1 (-), NZ_MUFR01000101.1 (-), NZ_MUFR01000091.1 (-), NZ_MUFR01000037.1 (+), NZ_MUFR01000052.1 (-), NZ_MUFR01000053.1 (-), NZ_MUFR01000068.1 (+), NZ_MUFR01000044.1 (-), NZ_MUFR01000005.1 (-), NZ_MUFR01000087.1 (+), NZ_MUFR01000039.1 (-), NZ_MUFR01000023.1 (+), NZ_MUFR01000036.1 (-), NZ_MUFR01000116.1 (+), NZ_MUFR01000085.1 (+), NZ_MUFR01000075.1 (-), NZ_MUFR01000035.1 (+), NZ_MUFR01000045.1 (-), NZ_MUFR01000020.1 (-), NZ_MUFR01000071.1 (-), NZ_MUFR01000049.1 (-), NZ_MUFR01000065.1 (+), NZ_MUFR01000056.1 (+), NZ_MUFR01000054.1 (+), NZ_MUFR01000022.1 (-), NZ_MUFR01000011.1 (+), NZ_MUFR01000048.1 (-), NZ_MUFR01000006.1 (-), NZ_MUFR01000019.1 (-), NZ_MUFR01000047.1 (-), NZ_MUFR01000041.1 (+), NZ_MUFR01000042.1 (+), NZ_MUFR01000014.1 (-), NZ_MUFR01000009.1 (-), NZ_MUFR01000010.1 (+), NZ_MUFR01000072.1 (-), NZ_MUFR01000024.1 (+), NZ_MUFR01000069.1 (+), NZ_MUFR01000027.1 (-), NZ_MUFR01000029.1 (-), NZ_MUFR01000018.1 (-) | NZ_MUFR01000077.1 (-), NZ_MUFR01000057.1 (-), NZ_MUFR01000055.1 (+), NZ_MUFR01000046.1 (+), NZ_MUFR01000008.1 (-), NZ_MUFR01000021.1 (-), NZ_MUFR01000038.1 (-), NZ_MUFR01000003.1 (+), NZ_MUFR01000034.1 (-), NZ_MUFR01000061.1 (+), NZ_MUFR01000031.1 (-), NZ_MUFR01000026.1 (+), NZ_MUFR01000084.1 (-), NZ_MUFR01000062.1 (+), NZ_MUFR01000033.1 (+), NZ_MUFR01000017.1 (-), NZ_MUFR01000058.1 (+), NZ_MUFR01000066.1 (-), NZ_MUFR01000032.1 (-) | NZ_MUFR01000064.1, NZ_MUFR01000088.1, NZ_MUFR01000102.1, NZ_MUFR01000103.1, NZ_MUFR01000104.1, NZ_MUFR01000105.1, NZ_MUFR01000106.1, NZ_MUFR01000107.1, NZ_MUFR01000108.1, NZ_MUFR01000109.1, NZ_MUFR01000110.1, NZ_MUFR01000111.1, NZ_MUFR01000112.1, NZ_MUFR01000113.1, NZ_MUFR01000114.1, NZ_MUFR01000115.1, NZ_MUFR01000117.1, NZ_MUFR01000118.1, NZ_MUFR01000119.1, NZ_MUFR01000120.1, NZ_MUFR01000121.1, NZ_MUFR01000122.1, NZ_MUFR01000123.1, NZ_MUFR01000124.1, NZ_MUFR01000125.1, NZ_MUFR01000126.1, NZ_MUFR01000127.1, NZ_MUFR01000128.1, NZ_MUFR01000129.1, NZ_MUFR01000130.1, NZ_MUFR01000131.1, NZ_MUFR01000132.1, NZ_MUFR01000133.1, NZ_MUFR01000134.1, NZ_MUFR01000135.1, NZ_MUFR01000136.1, NZ_MUFR01000137.1, NZ_MUFR01000138.1, NZ_MUFR01000139.1, NZ_MUFR01000140.1, NZ_MUFR01000141.1, NZ_MUFR01000142.1, NZ_MUFR01000143.1, NZ_MUFR01000144.1, NZ_MUFR01000145.1, NZ_MUFR01000146.1, NZ_MUFR01000147.1, NZ_MUFR01000148.1, NZ_MUFR01000149.1, NZ_MUFR01000150.1, NZ_MUFR01000151.1, NZ_MUFR01000152.1, NZ_MUFR01000153.1, NZ_MUFR01000154.1, NZ_MUFR01000155.1, NZ_MUFR01000156.1, NZ_MUFR01000157.1, NZ_MUFR01000158.1, NZ_MUFR01000159.1, NZ_MUFR01000160.1, NZ_MUFR01000161.1, NZ_MUFR01000162.1, NZ_MUFR01000163.1, NZ_MUFR01000164.1, NZ_MUFR01000165.1, NZ_MUFR01000166.1, NZ_MUFR01000167.1, NZ_MUFR01000168.1, NZ_MUFR01000169.1, NZ_MUFR01000170.1, NZ_MUFR01000171.1, NZ_MUFR01000172.1, NZ_MUFR01000173.1, NZ_MUFR01000174.1, NZ_MUFR01000175.1, NZ_MUFR01000176.1, NZ_MUFR01000177.1, NZ_MUFR01000178.1, NZ_MUFR01000179.1, NZ_MUFR01000180.1, NZ_MUFR01000181.1, NZ_MUFR01000182.1, NZ_MUFR01000183.1, NZ_MUFR01000184.1, NZ_MUFR01000185.1, NZ_MUFR01000186.1, NZ_MUFR01000187.1, NZ_MUFR01000188.1, NZ_MUFR01000189.1, NZ_MUFR01000190.1, NZ_MUFR01000191.1, NZ_MUFR01000192.1, NZ_MUFR01000193.1, NZ_MUFR01000194.1, NZ_MUFR01000195.1, NZ_MUFR01000196.1, NZ_MUFR01000197.1, NZ_MUFR01000198.1, NZ_MUFR01000199.1, NZ_MUFR01000200.1, NZ_MUFR01000201.1, NZ_MUFR01000202.1, NZ_MUFR01000203.1, NZ_MUFR01000204.1, NZ_MUFR01000205.1, NZ_MUFR01000206.1, NZ_MUFR01000207.1, NZ_MUFR01000208.1, NZ_MUFR01000209.1, NZ_MUFR01000210.1, NZ_MUFR01000211.1, NZ_MUFR01000212.1, NZ_MUFR01000213.1, NZ_MUFR01000214.1, NZ_MUFR01000215.1, NZ_MUFR01000216.1, NZ_MUFR01000217.1, NZ_MUFR01000218.1, NZ_MUFR01000219.1, NZ_MUFR01000220.1, NZ_MUFR01000221.1, NZ_MUFR01000222.1, NZ_MUFR01000223.1, NZ_MUFR01000224.1, NZ_MUFR01000225.1, NZ_MUFR01000226.1, NZ_MUFR01000227.1, NZ_MUFR01000228.1, NZ_MUFR01000229.1, NZ_MUFR01000230.1, NZ_MUFR01000231.1, NZ_MUFR01000232.1, NZ_MUFR01000233.1, NZ_MUFR01000234.1, NZ_MUFR01000235.1, NZ_MUFR01000236.1, NZ_MUFR01000237.1, NZ_MUFR01000238.1, NZ_MUFR01000239.1, NZ_MUFR01000240.1, NZ_MUFR01000241.1, NZ_MUFR01000242.1, NZ_MUFR01000243.1, NZ_MUFR01000244.1, NZ_MUFR01000245.1, NZ_MUFR01000246.1, NZ_MUFR01000247.1, NZ_MUFR01000248.1, NZ_MUFR01000050.1, NZ_MUFR01000067.1, NZ_MUFR01000074.1, NZ_MUFR01000078.1, NZ_MUFR01000079.1, NZ_MUFR01000081.1, NZ_MUFR01000083.1, NZ_MUFR01000086.1, NZ_MUFR01000089.1, NZ_MUFR01000090.1, NZ_MUFR01000093.1, NZ_MUFR01000094.1, NZ_MUFR01000095.1, NZ_MUFR01000096.1, NZ_MUFR01000097.1, NZ_MUFR01000098.1, NZ_MUFR01000099.1, NZ_MUFR01000100.1 |
| *S. proteolyticus* DSM 19052^T^ | NZ_MUFP01000028.1 (-), NZ_MUFP01000003.1 (+), NZ_MUFP01000023.1 (-), NZ_MUFP01000030.1 (-), NZ_MUFP01000006.1 (+), NZ_MUFP01000012.1 (+), NZ_MUFP01000024.1 (-), NZ_MUFP01000029.1 (-), NZ_MUFP01000009.1 (-), NZ_MUFP01000040.1 (+), NZ_MUFP01000015.1 (-), NZ_MUFP01000033.1 (+), NZ_MUFP01000018.1 (+), NZ_MUFP01000035.1 (-), NZ_MUFP01000005.1 (-), NZ_MUFP01000010.1 (+), NZ_MUFP01000039.1 (-), NZ_MUFP01000026.1 (-), NZ_MUFP01000016.1 (+), NZ_MUFP01000042.1 (-), NZ_MUFP01000034.1 (+), NZ_MUFP01000048.1 (+), NZ_MUFP01000001.1 (+), NZ_MUFP01000011.1 (+), NZ_MUFP01000027.1 (-), NZ_MUFP01000007.1 (+), NZ_MUFP01000004.1 (+), NZ_MUFP01000038.1 (-), NZ_MUFP01000020.1 (-), NZ_MUFP01000036.1 (+), NZ_MUFP01000013.1 (-), NZ_MUFP01000017.1 (-) | NZ_MUFP01000008.1 (-), NZ_MUFP01000037.1 (+), NZ_MUFP01000032.1 (+), NZ_MUFP01000021.1 (+), NZ_MUFP01000025.1 (-), NZ_MUFP01000002.1 (-), NZ_MUFP01000019.1 (-), NZ_MUFP01000031.1 (+), NZ_MUFP01000022.1 (-), NZ_MUFP01000014.1 (+) | NZ_MUFP01000041.1, NZ_MUFP01000043.1, NZ_MUFP01000044.1, NZ_MUFP01000045.1, NZ_MUFP01000046.1, NZ_MUFP01000047.1, NZ_MUFP01000049.1, NZ_MUFP01000050.1, NZ_MUFP01000051.1 |
| *S. sharmensis* DSM 18182^T^ | NZ_MUFC01000028.1 (-), NZ_MUFC01000005.1 (+), NZ_MUFC01000026.1 (-), NZ_MUFC01000011.1 (+), NZ_MUFC01000002.1 (+), NZ_MUFC01000001.1 (-), NZ_MUFC01000012.1 (+), NZ_MUFC01000010.1 (-), NZ_MUFC01000008.1 (-), NZ_MUFC01000035.1 (-), NZ_MUFC01000014.1 (-), NZ_MUFC01000006.1 (+), NZ_MUFC01000032.1 (-), NZ_MUFC01000023.1 (+), NZ_MUFC01000003.1 (+), NZ_MUFC01000025.1 (-), NZ_MUFC01000017.1 (-), NZ_MUFC01000022.1 (-), NZ_MUFC01000019.1 (-), NZ_MUFC01000007.1 (+), NZ_MUFC01000020.1 (+), NZ_MUFC01000030.1 (-), NZ_MUFC01000016.1 (+), NZ_MUFC01000018.1 (+), NZ_MUFC01000021.1 (-), NZ_MUFC01000027.1 (-) | NZ_MUFC01000009.1 (-), NZ_MUFC01000015.1 (-), NZ_MUFC01000031.1 (+), NZ_MUFC01000004.1 (-), NZ_MUFC01000024.1 (+), NZ_MUFC01000013.1 (-) | NZ_MUFC01000029.1, NZ_MUFC01000033.1, NZ_MUFC01000034.1, NZ_MUFC01000036.1, NZ_MUFC01000037.1, NZ_MUFC01000038.1, NZ_MUFC01000039.1, NZ_MUFC01000040.1 |
| *S. siamensis* JCM 14472^T^ | NZ_MUFB01000036.1 (+), NZ_MUFB01000002.1 (+), NZ_MUFB01000043.1 (+), NZ_MUFB01000017.1 (-), NZ_MUFB01000048.1 (-), NZ_MUFB01000001.1 (-), NZ_MUFB01000046.1 (+), NZ_MUFB01000015.1 (+), NZ_MUFB01000003.1 (+), NZ_MUFB01000044.1 (+), NZ_MUFB01000019.1 (-), NZ_MUFB01000016.1 (+), NZ_MUFB01000030.1 (-), NZ_MUFB01000008.1 (+), NZ_MUFB01000038.1 (-), NZ_MUFB01000009.1 (+), NZ_MUFB01000018.1 (-), NZ_MUFB01000022.1 (+), NZ_MUFB01000035.1 (-), NZ_MUFB01000004.1 (+), NZ_MUFB01000055.1 (-), NZ_MUFB01000040.1 (+), NZ_MUFB01000041.1 (+), NZ_MUFB01000006.1 (-), NZ_MUFB01000045.1 (+), NZ_MUFB01000007.1 (-), NZ_MUFB01000058.1 (-), NZ_MUFB01000039.1 (-), NZ_MUFB01000021.1 (-), NZ_MUFB01000024.1 (-), NZ_MUFB01000025.1 (-), NZ_MUFB01000028.1 (+), NZ_MUFB01000027.1 (-), NZ_MUFB01000005.1 (+), NZ_MUFB01000049.1 (+), NZ_MUFB01000034.1 (-), NZ_MUFB01000029.1 (-), NZ_MUFB01000050.1 (+), NZ_MUFB01000013.1 (-), NZ_MUFB01000014.1 (+) | NZ_MUFB01000042.1 (+), NZ_MUFB01000010.1 (-), NZ_MUFB01000026.1 (-), NZ_MUFB01000047.1 (-), NZ_MUFB01000033.1 (-), NZ_MUFB01000011.1 (+), NZ_MUFB01000012.1 (+), NZ_MUFB01000023.1 (-), NZ_MUFB01000032.1 (+), NZ_MUFB01000031.1 (+), NZ_MUFB01000020.1 (+), NZ_MUFB01000037.1 (+) | NZ_MUFB01000051.1, NZ_MUFB01000052.1, NZ_MUFB01000053.1, NZ_MUFB01000054.1, NZ_MUFB01000056.1, NZ_MUFB01000057.1, NZ_MUFB01000059.1, NZ_MUFB01000060.1, NZ_MUFB01000061.1 |
